# Yeast 5 – an expanded reconstruction of the *Saccharomyces cerevisiae* metabolic network

**DOI:** 10.1186/1752-0509-6-55

**Published:** 2012-06-04

**Authors:** Benjamin D Heavner, Kieran Smallbone, Brandon Barker, Pedro Mendes, Larry P Walker

**Affiliations:** 1 Department of Biological & Environmental Engineering, Cornell University, Ithaca, NY, 14853, USA; 2 Manchester Centre for Integrative Systems Biology, University of Manchester, Manchester, M1 7DN, UK; 3 Department of Biological Statistics and Computational Biology, Cornell University, Ithaca, NY, 14853, USA; 4 School of Computer Science, University of Manchester, Manchester, M13 9PL, UK

**Keywords:** Metabolic, Reconstruction, Yeast, Flux balance analysis, GEM, GENRE, Model

## Abstract

**Background:**

Efforts to improve the computational reconstruction of the *Saccharomyces cerevisiae* biochemical reaction network and to refine the stoichiometrically constrained metabolic models that can be derived from such a reconstruction have continued since the first stoichiometrically constrained yeast genome scale metabolic model was published in 2003. Continuing this ongoing process, we have constructed an update to the Yeast Consensus Reconstruction, Yeast 5. The Yeast Consensus Reconstruction is a product of efforts to forge a community-based reconstruction emphasizing standards compliance and biochemical accuracy via evidence-based selection of reactions. It draws upon models published by a variety of independent research groups as well as information obtained from biochemical databases and primary literature.

**Results:**

Yeast 5 refines the biochemical reactions included in the reconstruction, particularly reactions involved in sphingolipid metabolism; updates gene-reaction annotations; and emphasizes the distinction between reconstruction and stoichiometrically constrained model. Although it was not a primary goal, this update also improves the accuracy of model prediction of viability and auxotrophy phenotypes and increases the number of epistatic interactions. This update maintains an emphasis on standards compliance, unambiguous metabolite naming, and computer-readable annotations available through a structured document format. Additionally, we have developed MATLAB scripts to evaluate the model’s predictive accuracy and to demonstrate basic model applications such as simulating aerobic and anaerobic growth. These scripts, which provide an independent tool for evaluating the performance of various stoichiometrically constrained yeast metabolic models using flux balance analysis, are included as Additional files 1, 2 and 3.

**Conclusions:**

Yeast 5 expands and refines the computational reconstruction of yeast metabolism and improves the predictive accuracy of a stoichiometrically constrained yeast metabolic model. It differs from previous reconstructions and models by emphasizing the distinction between the yeast metabolic reconstruction and the stoichiometrically constrained model, and makes both available as Additional file 4 and Additional file 5 and at http://yeast.sf.net/ as separate systems biology markup language (SBML) files. Through this separation, we intend to make the modeling process more accessible, explicit, transparent, and reproducible.

## Background

Efforts to improve the computational reconstruction of the *Saccharomyces cerevisiae* biochemical reaction network and to refine the metabolic models that can be derived from such a reconstruction have continued since the first yeast genome scale metabolic model was published [1]. The distinction between reconstruction (termed GEnome scale Network REconstructions (GENREs) [2]) and derived models (termed GEnome scale Models (GEMs) [3]) remains important to differentiate between the established biochemical knowledge included in a GENRE and the modeling assumptions required for analysis or simulation with a GEM. A GENRE serves as a structured knowledge base of established biochemical facts, while a GEM is a model which supplements the established biochemical information with additional (potentially hypothetical) information to enable computational simulation and analysis. Examples of widely used yeast GENREs include the Kyoto Encylopedia of Genes and Genomes, KEGG [4], and the Yeast Biochemical Pathway Database, YeastCyc [5]. The history of yeast GEMs has recently been reviewed [6].

Though a GEM may be considered finished when it is sufficient for a particular modeling application, the effort to build a complete and accurate GENRE is ongoing as biochemical research continues (even information that is fundamental to the construction of a GENRE, such as genome annotation, is considered to be a working hypothesis and subject to ongoing revision [7]). Reflecting the ongoing process of yeast GEM and GENRE improvement [6], we have constructed an update to the Yeast Consensus Reconstruction [8]. The Yeast Consensus Reconstruction is a product of efforts to forge a community-based reconstruction emphasizing standards compliance and biochemical accuracy via evidence-based selection of reactions. It draws upon models published by a variety of independent research groups [1,9-12], as well as information obtained from biochemical databases and primary literature. Thus, the Yeast Consensus Reconstruction serves as an example of the community-based approach which has given rise to the concept of a “reconstruction annotation jamboree” [13]. Though there remain many challenging problems to implementing and maintaining community-based science [14], the jamboree approach to network reconstruction and model building has also been successfully applied to build a consensus reconstruction and model of *Salmonella* Typhimurium LT2 [15].

The Yeast Consensus Reconstruction has benefited from the continued involvement of the broader research community. Previous updates to the Yeast Consensus Reconstruction [16] have focused on filling gaps in the metabolic reconstruction to improve network connectivity in a graph-theoretical sense, expanding the reconstruction of portions of metabolism that had not been included in previous reconstructions, and enabling Flux Balance Analysis (FBA) [17] by adding the necessary (but hypothetical) transport reactions and sink reactions (such as the biomass reaction). These low-confidence reactions in the Yeast Consensus Reconstruction are annotated with use of specialized Systems Biology Ontology (SBO) terms [18], an approach designed to facilitate differentiation between the higher-confidence reactions which form the Yeast GENRE and the lower confidence reactions required to evaluate a GEM with FBA. Enabling FBA of the consensus reconstruction has resulted in increased interest in applying the model to guide bioengineering efforts [19,20]. In turn, this increased interest has stimulated community participation, which has highlighted opportunities for further improving the Consensus Yeast Reconstruction GENRE and the derived GEM.

Therefore, we decided to undertake an update to the Yeast Consensus Reconstruction to refine the biochemical reactions included in the GENRE, particularly reactions involved in sphingolipid metabolism [20,21]; to review gene-reaction annotation; to emphasize and clarify the distinction between GENRE and GEM; to facilitate application of the GEM for bioengineering applications; and to solicit and facilitate further collaboration among researchers who wish to further improve the yeast GENRE and GEM. Although it was not a primary goal, this update also improves the accuracy of GEM phenotype predictions due to the incorporation of reaction constraints from previous models and relevant literature. We endeavored to conduct this update while maintaining an emphasis on standards compliance, unambiguous metabolite naming, and computer-readable annotations available through a structured document format. The metabolites included in Yeast 5 are unambiguously annotated with their identifiers in the Chemical Entitites of Biological Interest (ChEBI) database [22], and reactions are annotated with the PubMed ID of primary literature evidence justifying the reaction’s inclusion in the reconstruction.

We have incorporated the results of these efforts to the consensus reconstruction to produce Yeast 5. Yeast 5 expands and refines the yeast GENRE and improves the predictive accuracy of the yeast GEM. Further, it differs from previous reconstructions and models by emphasizing the distinction between the yeast GENRE and GEM, and makes both available as separate systems biology markup language (SBML) files [23]. Through this separation of GENRE and GEM, we intend to make the modeling process more explicit, transparent, and reproducible. Both files are available from YeastNet (http://yeast.sf.net/). In addition to the GEM and GENRE SBML files, we have developed MATLAB scripts to evaluate the model’s predictive accuracy and to demonstrate basic model applications, such as simulating aerobic and anaerobic metabolism with Yeast 5. These scripts, which provide an independent tool for evaluating the performance of various yeast GEMs, are included as additional files.

## Results

### Improvements to facilitate community use and collaboration

As a consensus reconstruction and model, Yeast 5 will depend upon community use and suggested modifications for future improvement. The Yeast 5 GENRE may serve as a resource for construction of new models, of both genome and smaller scale. Thus, in addition to the emphasis on standards compliance, the Yeast 5 SBML files include coding conventions to facilitate use by popular software. Recognizing that MATLAB is a commonly used platform for systems and computational biology [24], we include scripts demonstrating use of both the GENRE and GEM with the SBML Toolbox [25] and the COBRA Toolbox [26]. These MATLAB scripts, testYeastModel.m, modelToReconstruction.m, and fluxDistribution.m, are available as Additional files 1,2 and 3. Additionally, as discussed in Materials and Methods, Yeast 5 includes conventions for exchange reactions and boundary species which are used in the MATLAB-compatible COBRA Toolbox, although such conventions are not currently included in SBML specifications [23].

### Yeast GENRE changes

The Yeast 5 GENRE is an evidence-driven biochemical knowledge-base. It does not include the low-confidence or hypothetical reactions and metabolites required to conduct FBA, nor does it include constraints on reaction reversibility which may be added in the course of model-building. Since it does not include compounds such as “biomass”, reactions such as “growth”, or hypothetical intercompartmental transport reactions, the Yeast 5 GENRE is more specific than Yeast 4, which did not differentiate between GENRE and GEM. It contains 1418 metabolites which participate in 2110 reactions, catalyzed by 918 verified *Saccharomyces cerevisiae* genes. In comparison, Yeast 4 includes 1481 metabolites, 2030 reactions, and 924 genes. The Yeast 5 GENRE does not include genes annotated in the Saccharomyces Genome Database (SGD) as “dubious” or “uncharacterized”, while Yeast 4 includes 4 such genes (YFR055W, YML082W, YPL275W, and YPL276W). Yeast 4 included reactions annotated with 29 open reading frames which are not included in the Yeast 5 GENRE. However, the Yeast 5 GENRE includes 23 open reading frames which are not included in Yeast 4. The 29 open reading frames which are present in Yeast 4 but not Yeast 5 are: YAL014C, YAL030W, YAR042W, YCR073W-A, YDL019C, YDR313C, YDR331W, YDR468C, YEL011W, YEL013W, YER093C, YFR055W, YGR199W, YHR005C, YHR073W, YMR068W, YNL006W, YNR034W, YOL078W, YPL145C, YPL275W, YPL276W, YOR237W, YIL105C, YJL058C, YJR160C, YKL203C, YML082W, and YNL047C. These open reading frames were removed from reaction annotations because of inadequate literature evidence supporting the Yeast 4 annotation. The 23 open reading frames included in the Yeast 5 GENRE but not in Yeast 4 are: YBR001C, YBR058C-A, YBR161W, YBR199W, YDR196C, YDR367W, YGR138C, YGR277C, YMR241W, YMR278W, YMR298W, YPL023C, YPL053C, YPL189W, YPR156C, YOR175C, YGL084C, YIL083C, YJL200C, YKL088W, YKL132C, YML056C, and YNL029C. Additional annotation information about these ORFs is provided in Additional file 6: Table S1 (ORFs present in Yeast 4 but not in Yeast 5) and Additional file 7: Table S2 (ORFs present in Yeast 5 but not in Yeast 4).

In addition to being more specific, the Yeast 5 GENRE is also more complete than Yeast 4. Sphingolipid metabolism has been acknowledged to be a poorly reconstructed portion of the yeast metabolic network since the first yeast GEM, iFF708 [1]. Yeast 5 incorporates suggested literature-referenced refinements to sphingolipid metabolism [20,21]. Thus the Yeast 5 GENRE contains the most complete reconstruction to date of the broad suite of complex sphingolipids that has been observed in yeast [27].

### Yeast GEM changes

The Yeast 5 GEM includes biomass demand functions and low-confidence reactions such as intercompartmental transport reactions which enhance network connectivity and enable FBA. It also includes reaction directionality constraints to improve the accuracy of model phenotype predictions and exchange reactions which allow model users to simulate a growth medium. The Yeast 5 GEM includes more reactions and metabolites than previously published yeast GEMs (Table1), though it includes 6 fewer genes than Yeast 4. The Yeast 5 GEM includes reactions annotated with 918 different open reading frames, accounting for 18.5% of the 4949 verified open reading frames included in the Saccharomyces Genome Database [28] as of October 12, 2011. The Yeast 5 GEM has 326 more directionally constrained reactions than Yeast 4 (69% of all reactions in the Yeast 5 GEM are constrained, compared to 55.6% in Yeast 4). The majority of these new constraints are applied to reactions involved in cofactor utilization or production, with a particular emphasis on reactions involving ATP/ADP. Reactions involving NAD(P)/H are constrained where literature evidence supports irreversible reactions *in vivo.*

**Table 1:** Comparison of yeast metabolic models

	**Yeast 5**	**Yeast 4**^**a**^	**iMM904bs**^**b**^	**iND750**^**c**^
**Model description**				
Number of metabolites	1655	1481	1228	1061
Number of reactions	2110	2030	1575	1266
Number of genes	918	924	904	750
Number of dubious genes^d^	0	4	17	17
Blocked reactions^e^	38%	26%	31%	41%
**Viability analysis**				
Sensitivity^f^	97%	95%	93%	96%
Specificity^g^	47%	44%	57%	43%
Positive predictive value^h^	86%	85%	89%	87%
Negative predictive value^i^	84%	73%	69%	77%
Geometric mean^j^	46%	42%	53%	41%
**Auxotrophy analysis**				
Auxotroph-inducing genes included^k^	70	73	73	69
Correct auxotroph predictions	73%	66%	69%	58%
Incorrectly predicted as viable in minimal media	24%	30%	27%	39%
Incorrectly predicted as inviable in supplemented media	3%	4%	4%	3%
**Epistatic interaction analysis**^**l**^				
Epistatic interations (% of pairwise genes)	16%	-	15%	21%
Total number of epistatic interactions	65,730	-	63,176	57,808
Average Additional Interactions per additional gene^m^	196.46	-	34.63	-

^a^yeast 4: [16].

^b^iMM904bs: [29].

^c^iND750: [9].

^d^dubious genes are ORFs annotated as "dubious" (809 ORFs) or "uncharacterized" (857 ORFs) in SGD.

^e^blocked reactions cannot carry flux.

^f^True positive/(true positive + false negative).

^g^True negative/(true negative + false positive).

^h^True positive/(true positive + false positive).

^i^True negative/(true negative + false negative).

^j^see [10] for discussion of applying geometric mean

^k^see Additional file 8: Table S3 and Additional file 9: Table S4 for list of essential genes and auxotroph-inducing genes.

^l^At 50%x50% flux reductions. See Additional file 10: Table S5 for other restriction levels.

^m^ number of interactions/number of genes, compared to iND750 as base case.

Since the Yeast GEM includes both general classes of compounds (e.g.“fatty acid”) and specific members of these classes (e.g. “octanoate”), we use non-reversible encapsulating reactions called “isa” reactions to provide pathways from specific to generic compounds (e.g., octanoate “isa” fatty acid). The use of “isa” reactions is discussed further in the Discussion section. To accommodate the more specific biochemistry included in the Yeast 5 GENRE, the Yeast 5 GEM includes 261 “isa” reactions, compared to 162 in Yeast 4. Additionally, the Yeast 5 GEM includes 2 different lipid pseudoreactions, which create the “lipid” portion of biomass (details of the Yeast 5 GEM biomass definition are included as Additional file 11: Table S6). As described in the “simulating yeast growth” discussion, including two differing biomass definitions enables simulation of anaerobic yeast metabolism despite the incomplete reconstruction of yeast lipid biochemistry in the Yeast 5 GENRE.

### Yeast GEM performance

Though improving gene essentiality predictions was not a primary objective of updating Yeast 4, simulations using Yeast 5 have increased agreement with a list of essential genes and genes which cause auxotrophies; more realistic prediction of internal fluxes; and increased number of genetic interactions. Additionally, simulations with the Yeast 5 GEM predict auxotrophies resulting from gene deletions better than other recent yeast GEMs, and predict gene essentiality with accuracy comparable to other recent yeast GEMs (Table1).

#### Gene essentiality predictions

Since the phenotype resulting from a gene mutation is dependent upon media and environmental conditions as well as changes to the metabolic network, the use of gene essentiality predictions as a metric for model evaluation requires careful definition of both simulation assumptions and of the data set used for comparison between simulation and observation. This is particularly important if such a metric is to be used for comparison of different GEMs. We document our approach to simulating gene essentiality in Materials and Methods, and in the testYeastmodel.m MATLAB script included as supplemental material.

The Yeast 5 GEM includes reactions annotated with 918 genes. 144 of these genes are included in a list of essential genes we compiled from the Saccharomyces Genome Deletion project [30] and annotation included in Saccharomyces Genome Database [28] ( Additional file 8: Table S3). An additional 70 genes are included in a list of genes causing auxotrophies when deleted ( Additional file 9: Table S4). (The construction of these gene lists is described in Materials and Methods). The remaining 704 genes in the model are not on the compiled lists of essential or auxotroph-inducing genes, and are therefore considered inessential.

The results of single gene deletion simulations conducted via FBA of the Yeast 5 GEM using a simulated glucose-limited defined media are summarized in Table2. The model predicted that biomass could be produced in 684 of the 704 cases in which genes annotated as inessential or non-auxotrophic were deleted (true positive results) and that biomass could not be produced in 20 cases where these inessential or non-auxotrophic genes were deleted (false negative results). Thus, the model has a 97.2% sensitivity for this list of essential genes. If the model simulation predicted that biomass could be produced following a gene deletion, the deleted gene was not listed as essential or auxotroph-inducing in 86% of the cases (an 86% positive predictive value).

**Table 2:** Single-gene Deletion Results (918 genes)

**684 **(75%) True Positives (model simulation predicts growth when inessential genes are deleted)	**20 **(2%) False Negatives (model simulation predicts no growth when inessential genes are deleted)
**113** (12%) False Positives (model simulation predicts growth when essential genes are deleted)	**101** (11%) True Negatives (model simulation predicts no growth when essential genes are deleted)

The model predicted that biomass could not be produced following deletion of 101 of the of the 214 genes included on the essential or auxotrophy-inducing gene lists (true negative results), but that biomass could still be produced following deletion of 113 genes included on those lists (false positive results). Thus, the model has a 47% specificity for this list of essential genes. If the model simulation predicted that biomass could not be produced following a gene deletion, the deleted gene was listed as essential or auxotroph-inducing in 83.5% of the cases (an 83.5% negative predictive value).

Comparing Yeast 5 GEM knockout simulations with our list of essential and auxotroph-inducing genes yields a geometric mean overall predictive accuracy (as suggested by [10] of 45.88%, an improvement over Yeast 4’s 41.61% geometric mean accuracy for this gene list. These results, including comparison of the simulations using other recently published GEMs and the same essential and auxotroph-inducing gene lists, are summarized in Table1.

#### *Auxotroph-inducing mutations*

To extend our analysis of Yeast 5 GEM simulation capabilities, we conducted additional FBA growth simulation focusing on the 70 genes included in both the Yeast 5 GEM and the list of genes whose mutation or deletion causes auxotrophies. In 51 single-gene deletion simulations, the model predicted that biomass could not be produced in minimal media but could be produced in a supplemented media, the expected behavior for an auxotroph mutant (see Materials and Methods for more information about our approach to simulated media). In 17 cases, model simulation predicted that biomass could be produced in minimal media, and thus did not accurately predict the auxotrophic phenotype. In 2 cases, model simulation predicted that biomass could not be produced in either minimal or maximal media, and so the model incorrectly predicted that the gene deletion could not be saved by media supplementation.

Auxotroph phenotypes have not previously been a metric used to evaluate yeast GEMs. We found that simulation with the Yeast 5 GEM had better agreement with observed auxotrophic phenotypes than simulations conducted with other previously published GEMs (Table1).

#### *Flux predictions*

Optimal solutions found when conducting FBA of the Yeast 5 GEM to maximize biomass flux include fluxes through key internal reactions, a flux distribution which better matches fluxes observed *in vivo*[31] than solutions found when applying FBA to Yeast 4 (Table3). Specifically, applying FBA to the Yeast 5 GEM in a simulated glucose-limited aerobic environment predicts that the reactions of glycolysis and the TCA cycle have fluxes, and that ethanol is not produced. When the model constraints are adjusted to simulate an anaerobic environment, FBA predicts fluxes through the reactions of glycolysis, but not the TCA cycle, and ethanol is produced. Thus, simulations with the Yeast 5 GEM reflect the shift from respiratory to fermentative metabolism which is observed in oxygen-limited yeast cultures. Simulations using the Yeast 4 GEM do not reflect this same behavior.

**Table 3:** Yeast 5 and Yeast 4 simulated flux predictions

	**yeast 5**	**yeast 4**
**Sample FBA flux predictions**		
glucose-limited, aerobic growth rate	0.09	0.17
glucose-limited, anaerobic growth rate	0.02	0
aerobic flux through glycolysis^a^	1.33	0.89
anaerobic flux through glycolysis	1.85	-
aerobic flux through TCA cycle^b^	1.06	0.01
anaerobic flux through TCA cycle	0	-
aerobic ethanol production	0	0
anaerobic ethanol production	1.74	-

Fluxes are normalized to the glucose uptake flux, which is set to 1 mmol/g dry weight/h.

^a^glycolysis flux measured through pyruvate kinase.

^b^TCA cycle flux measured through malate dehydrogenase.

#### *Increased number of genetic interactions*

Recognizing recent efforts to investigate system-level organization of cellular metabolism via the phenotypic effects of multiple gene deletions using yeast GEMs [32-36], we compared the number of epistatic interactions predicted by growth simulations using the Yeast 5 GEM with the number of interactions predicted by simulations using the iMM904 [12] and iND750 [9] models (Table1). When reaction fluxes were restricted to 50% of wild-type in a pairwise manner, we found that a lower percentage of genes included in the Yeast 5 and iMM904 GEMs were predicted to exhibit epistatic interactions in FBA simulations, but the expanded size of these models led to an increased number of total epistatic interactions compared to the iND750 GEM. If the number of interactions are averaged over the number of genes in each model, the Yeast 5 GEM adds an average of 196.5 new epistatic interactions per additional gene, and iMM904 adds an average of 34.6 new interactions per gene. The number of interactions predicted using each of these models using varying levels of flux restriction for each gene and reaction pair are provided as Additional file 9: Table S4.

### Limitations

Research to expand our understanding of yeast metabolism and biochemistry is ongoing, and the process of integrating established biochemical knowledge into computational reconstructions lags research advancements. Thus, the Yeast 5 GENRE is not a complete reconstruction of the yeast biochemical network, and though it offers improvements over earlier models, simulations using the Yeast 5 GEM do not fully reflect observed biological phenomena. GENRE and GEM limitations suggest opportunities for future efforts to improve computational reconstruction of established biochemistry and can highlight portions of metabolism that are ripe for further research [37].

A limitation of the Yeast 5 GENRE which suggests future opportunities for improving the Yeast Reconstruction is that due to the lack of information about enzyme specificity or the metabolic significance of variation among similar chemical species, the Yeast 5 GENRE uses general classes of chemical compounds rather than the enumeration of many similar compounds. For example, Yeast 5 generalizes the many possible triglyceride compounds which may be synthesized from fatty acyl moieties of varying length [27] into a single model species, called “triglyceride”. Yeast 5 also includes similar generalized species for other compounds, particularly those involved in lipid and sterol metabolism. Though this approach is also followed by previous yeast GENREs and other metabolic pathway tools, expansion of such general species by differentiating among biochemically relevant species has been shown to be a successful approach to expanding computational reconstructions of metabolic networks [20]. The appropriate level of detail or generalization for metabolic (or biochemical) network reconstruction depends upon the intended use of a GEM, and would be expected to change in the future as our knowledge of enzyme specificity and the metabolic relevance of differences among similar chemical compounds advances.

As with other reconstructed metabolic networks, the continued existence of blocked pathways (Table1) highlights that our knowledge of yeast metabolism is incomplete. Such blocked reactions are an important tool for documenting portions of metabolism that would benefit from further research [37]. The Yeast 5 GENRE remains limited by knowledge gaps. Where our knowledge of intercompartmental transport of metabolites is limited, such gaps pose a particular challenge to FBA. Thus, the Yeast 5 GEM includes hypothetical transport reactions to better connect portions of the metabolic network that are unconnected in a graph-theoretical sense. Such hypothetical transport reactions are annotated with SBO term [18] SBO:397 (“omitted process”).

Optimal solutions found when conducting FBA on the Yeast 5 GEM may include fluxes that differ from those observed in yeast: we have found optimal solutions in which mitochondrial coenzyme A is synthesized *in situ* rather than transported from the cytoplasm, and model growth simulations incorrectly predict that yeast is not a pantothenate auxotroph or a nicotinic acid auxotroph in anaerobic conditions. Additionally, although the model predicts that biomass can only be produced anaerobically if the biomass definition is modified (see Materials and Methods), the reason that simulated anaerobic biomass production using an unmodified biomass definition is blocked is not because of the biological requirement of yeast fatty acid desaturate for oxygen. Instead, simulated anaerobic biomass production is blocked due to other, as yet unidentified limitations in the reconstruction of phospholipid and sterol biosynthesis. Refining the solution space to more closely match observed biological phenomena through improved reconstruction or expanded constraints remains an ongoing research effort for reconstructed metabolic networks.

Due to varying interpretations of experimental evidence, uncertainty of metabolic mechanism, and varying approaches taken as modelers work to reconstruct different portions of metabolism, it is likely that the Yeast 5 consensus reconstruction has additional limitations which will be discovered as it is used. These limitations provide opportunities for continued research to improve the computational reconstruction and simulation of the yeast biochemical network. Thus, though Yeast 5 consists of a more complete reconstruction and more accurate model of yeast metabolism than previous efforts, the goal of building a complete and accurate computational reconstruction of yeast metabolism must remain an ongoing community effort.

## Discussion

Yeast 5 is the most recent update to the consensus reconstruction of the yeast metabolic network. It consists of a genome-scale reconstruction (GENRE), a genome scale model (GEM), and MATLAB scripts designed to facilitate evaluation of yeast GEMs and to demonstrate simulation and analysis using the COBRA and SBML toolboxes. This update improves the consensus reconstruction’s coverage of established biochemical knowledge, and improves the predictive ability of simulations using the yeast GEM. The included scripts lower the barriers for the research community to use the model and to contribute to the collaborative effort to improve the computational reconstruction of yeast metabolism.

### Models and reconstructions

We emphasize the distinction between a reconstruction, or GENRE, and a model, or GEM, to more clearly delineate the established biochemical knowledge of a reconstruction from the assumptions and hypotheses that are required for modeling and simulation. This distinction makes the modeling process more transparent and reproducible, essential attributes for community-based scientific efforts such as the consensus reconstruction of yeast metabolism.

### Evaluating yeast reconstructions and models

As evidence of improvements to the consensus yeast reconstruction, we have presented a comparison of viability, auxotrophy predictions, and genetic interaction effects, produced using FBA of the Yeast 5 GEM and other metabolic models (Table1). However, we emphasize that such metrics of phenotype predictive ability must be evaluated with great care. This need for careful use of such metrics has been discussed previously [38], but we have found that this point deserves additional emphasis. Specifically, the two goals of expanding the reconstruction of metabolic networks and improving model predictions of mutant viability may be contradictory in some situations. The Yeast 5 GEM sensitivity, specificity, positive predictive value, negative predictive value, and geometric mean could all be improved by the reduction of false positive predictions - simulations which predict that biomass can be produced although reactions annotated as being catalyzed by “essential genes” have been blocked. The number of false positive predictions could be reduced by expanding the biomass function to require products of reactions annotated with essential genes, by removing parallel pathways to force fluxes through reactions annotated with essential genes, or by removing metabolites and reactions which create dead-end pathways which include reactions annotated with essential genes (a method used to improve lethality prediction metrics when the iLL672 model was derived from iFF708 [10]). However, while such techniques improve a model’s ability to predict single-gene mutant viability, they also reduce the scope of a GENRE as a structured knowledge base of established biochemical facts.

Expanding the reconstruction of a metabolic network would be expected to increase both dead-end pathways and network redundancy. Dead ends would be introduced through the inclusion of established knowledge regarding pathways that are not fully elucidated. In such pathways, the production of intermediates may be established, but their fate is not yet known. In such cases, expanding the reconstruction of established knowledge would not be expected to improve a model’s ability to predict single-gene mutant viability, and so such metrics would not reflect the expanded scope of the reconstruction. Another example of network expansion which may not be reflected in single-gene deletion metrics is expanding the reconstruction’s coverage of isoenzymes. Network redundancy increases through expanded inclusion of isoenzymes, which introduce parallel metabolic paths for the production of chemical intermediates or products. In the absence of regulatory constraints (which are beyond the scope of a metabolic reconstruction), these parallel pathways would increase the rate of false positive prediction since a metabolic pathway from substrate to product would exist in the reconstruction, even if a given isoenzyme were individually insufficient to support growth *in vivo*. Thus, a metabolic model based upon a reconstruction with improved coverage of established biochemistry of isoenzymes would make less accurate predictions of individual gene essentiality than a model based upon a less complete reconstruction.

A second problem with metrics based upon lists of essential genes is that gene essentiality is dependent upon strain, media, and environmental conditions (for example, [39] identify mutants which are inositol auxotrophs only at elevated temperatures). Though a general definition of “essential” could imply “in complex media at 30 °C”, the difficulties of computationally reconstructing complex media and the lack of integration of temperature effects on metabolic networks means that there remains an element of subjectivity in defining a list of essential genes. Researchers have previously used different data sets to define gene essentiality for model analysis [9,12]. Thus, if model predictivity metrics are to be used, care must be taken to ensure a common list of essential genes when evaluating different models by such metrics. Yeast 5 includes the MATLAB script testYeastModel.m to document the list of genes we considered essential for our comparison of yeast GEMs.

That essentiality metrics must be used with care and considered in context is not to say that such metrics are without value, however. Model simulation results that differ from *in vivo* experiments can guide efforts to improve computational reconstruction or to highlight the need for additional biochemical investigation of metabolic dead ends. Simulations resulting in false negative results, in which the model predicts that biomass cannot be formed, but *in vivo* experiments have observed growth, suggest that the reconstruction is incomplete or the model has limitations such as incorrect biomass definition or missing simulated media components. Indeed, the use of gap filling algorithms to improve phenotype predictive metrics for metabolic models by adding hypothesized gene functions or reactions is considered standard practice for GEM development [40].

Like auxotroph phenotype predictions, predicted epistatic interactions have not previously been used as a metric for comparing yeast GEMs. And like other metrics based upon phenotypic prediction, the use of epistatic interactions must also be qualified. Specifically, expansion in the number of reactions in the model is a likely contributor to the amount of simulated epistasis. The number of genetic interactions can also be increased by pleiotropy, or the number of reactions associated to a particular gene. Yeast 5 has an 8.43% increase in the mean number of reactions per gene compared to iMM904. The most prominent example is ISC1, a gene important in sphingolipid metabolism. ISC1 is included in annotation for 60 reactions in Yeast 5, but only 18 in iMM904. ISC1 accounts for a 1% increase in pleiotropy by itself. FOX2, a multi-function enzyme involved in beta-oxidation, ranked highest for pleiotropy in iMM904 with 23 reactions. It is also associated with 23 reactions in Yeast 5. Additional trends in positive or negative epistasis across different types of mutations can also be observed for these models ( Additional file 10: Table S5) [41].

### Generic demand reactions in yeast 5 - towards a functional biomass definition

The yeast consensus reconstruction draws upon data sources with varying levels of compound specificity. For example, the KEGG database includes a general representation of yeast sphingolipid metabolism, while recent suggestions for changes to Yeast 4 include more specific chemical species [20]. Thus, some Yeast 5 reactions which are derived from KEGG use generic species as substrates or products, while reactions derived from other sources use more specific species. To accommodate the formation of generic chemical species for reactions which consume them while preserving biochemical accuracy in reactions that have more specific biochemistry, the Yeast GEM includes “isa” reactions. Examples of generic species produced by “isa” reactions include “complex sphingolipid”, “fatty acid”, and “acyl-CoA”.

Where “isa” reactions are reversible, they can introduce unrealistic interconversion of metabolites. For example, since octanoate is a fatty acid and hexadecanoate is a fatty acid, reversible “isa” reactions in a model would create a nonrealistic pathway by which octanoate could be converted to hexadecanoate via the “isa” reaction, instead of through biochemical pathways which have been documented *in vivo*. In order to prevent such non-realistic interconversion fluxes, Yeast 5 “isa” reactions are not reversible. Thus, more general compounds can only serve as sinks of more specific compounds in the Yeast 5 GEM, and not as sources.

**Figure 1:**
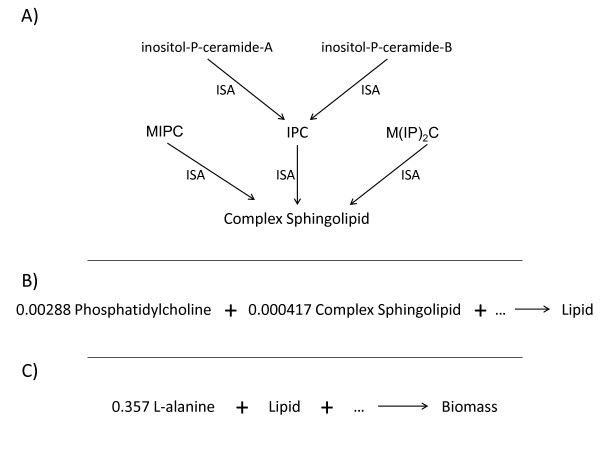
**Using “isa” reactions.** Yeast 5 uses “isa” reactions to encapsulate specific chemical species within more general classes. For example, **A)** the specific species inositol-P-ceramide-A “isa” inositol phosphoceramide (IPC). In turn, IPC “isa” complex sphingolipid. **B)** Complex sphingolipids participate in the stoichiometrically constrained reaction which produces the species “lipid”. **C)** The lipid species is a component of biomass. This hierarchical model structure embeds logic in the biomass definition: biomass consists of L-alanine AND phosphatidylcholine AND (inositol-P-ceramide-A OR Inositol-P-ceramide-B OR any of the 88 other complex sphingolipids included in the reconstruction). A model user is free to constrain the fluxes which produce specific complex sphingolipids to model an observed lipid composition, or may leave the model unconstrained if the more general biomass definition is sufficient for their needs

An unanticipated result of this approach to varying levels of biochemical specificity in the model is that “isa” reactions effectively embed the logical OR into the model. Thus, where the biomass definition includes “lipid” as a required component, this objective function can be satisfied by any of the compounds that can be converted to “lipid” via an “isa” reaction (Figure1). Thus, although “biomass” must be defined if maximizing biomass production is the objective function for FBA, “biomass” does not need to be a specifically determined compound with a fixed stoichiometry for FBA to be successfully applied to stoichiometrically constrained metabolic reconstructions.

### Continuing efforts to reconstruct yeast metabolism - an invitation for continued community involvement

Computational reconstruction and modeling of yeast metabolism is an ongoing project. Suggestions for improving the yeast consensus reconstruction or derived models should be submitted to network.reconstruction@manchester.ac.uk. Metabolites and enzymes should be unambiguously identified, using existing model or database (ChEBI or UniProt) identifiers. New reactions should be supplied with primary evidence for their mechanism and catalysis, via PubMed identifiers. Reactions without evidence should have clear reasons for their proposed addition.

We also invite researchers to submit models derived from the yeast consensus reconstruction for hosting at http://yeast.sf.net/. Assumptions and constraints should be documented, for example with code documenting how to build the GEM from the Yeast GENRE. Such models may be submitted for publication separately from updates to the Yeast GENRE, and may follow the iNNXXX naming convention which has been previously used for identifying GEMs [42].

## Conclusions

The Yeast 5 expansion of the Yeast Consensus Reconstruction refines the computational reconstruction of yeast metabolism and improves the predictive accuracy of a stoichiometrically constrained yeast metabolic model. It refines the biochemical reactions included in the reconstruction, particularly reactions involved in sphingolipid metabolism; updates gene-reaction annotations; and emphasizes the distinction between reconstruction (GENRE) and stoichiometrically constrained model (GEM). This update also improves the accuracy of model prediction of viability and auxotrophy phenotypes and increases the number of epistatic interactions. Yeast 5 differs from previous reconstructions and models by emphasizing the distinction between the yeast metabolic reconstruction and the stoichiometrically constrained model, and makes both available as Additional file 4 and Additional file 5 and at http://yeast.sf.net/ as separate systems biology markup language (SBML) files. Through this separation, we intend to make the modeling process more accessible, explicit, transparent, and reproducible. The Yeast Consensus Reconstruction remains a community-based resource which emphases standards compliance and biochemical accuracy via evidence-based selection of reactions.

Though Yeast 5 consists of a more complete reconstruction and more accurate model of yeast metabolism than previous efforts, the goal of building a complete and accurate computational reconstruction of yeast metabolism must remain an ongoing community effort. As Yeast 5 limitations are identified, they provide opportunities for continued research to improve the computational reconstruction and simulation of the yeast biochemical network.

## Methods

### Yeast 5 scope

The scope of Yeast Consensus Reconstruction was originally determined by the data sets used for its construction: the iMM904 [12] and iLL672 [10] models, which included information from the KEGG and SGD databases, along with other sources. Subsequently, the Consensus Reconstruction was expanded [16] to include information (particularly focusing on lipid metabolism) from the iIN800 model [11]. Yeast 5 further expands the scope of reconstruction to refine details of sphingolipid metabolism [20,21]. Though the stoichiometrically constrained approach can theoretically be expanded to include all biochemical reactions in the organism being modeled [43], the Yeast reconstruction is currently limited to the yeast metabolic network. Although Yeast 5 is not strain-specific, the auxotroph information we used for our analysis focused on auxotrophies documented in *S. cerevisiae* reference strain SC288C. Since multiple yeast genome sequences are now available, future updates to the Yeast Consensus Reconstruction may become strain specific.

### Deriving reconstruction (GENRE) from model (GEM)

We must maintain a distinction between the *reconstruction* of yeast metabolism, an evidence-driven biochemical knowledge-base, and its corresponding *model*, which relies on a number of assumptions to make quantitative flux predictions [40]. We discriminate between the two through the use of the Systems Biology Ontology (SBO) [18]. Specifically, reactions marked up with specific SBO terms may be automatically removed from a model to create a reconstruction. Encapsulating “isa” reactions of the form “A isa B” are annotated with SBO:395 (“encapsulating process”). Other reactions without literature evidence, that are omitted in the reconstruction, such as biomass production and most transport reactions without an associated transporter are annotated with SBO:397 (“omitted process”). Reaction constraints (lower and upper bounds) are also removed in the reconstruction. The transformation is performed using the SBMLToolbox [25].

### Yeast model conventions

The Yeast 5 GEM includes conventions to facilitate model analysis using the COBRA Toolbox, such as standardized representation of exchange reactions, though such conventions are not required in the SBML specification. However, we have chosen to rely upon the SBML standard for encoding reaction and metabolite annotation, rather than the nonstandard custom notes field currently used by the COBRA toolbox. This information, which includes metabolite ChEBI identifiers and literature references supporting reaction inclusion, is encoded in the model .sbml file and is available to MATLAB users via the SBML toolbox. An additional model convention is that the Yeast 5 GEM includes biomass as a species in the model to help make our approach to simulating biomass production more explicit.

Exchange reactions in the Yeast 5 GEM follow a convention of using compounds in the model as reactants, leading to exchange reactions of the form “reactant ->”, with an entry of +1 in the stoichiometric matrix. Thus, positive flux values for exchange reactions represent compounds produced in FBA simulation, and negative flux values represent compounds consumed. Since reactions must include both substrate and product in SBML, we have followed the COBRA toolbox convention of denoting exchange reaction species which lay outside the model with the subscript “_b”. These species are not loaded into the COBRA toolbox data structure, and serve only as placeholders for exchange reaction substrates.

We have chosen to include biomass as a species in the Yeast 5 GEM. Thus, when conducting FBA on the Yeast 5 GEM, the biomass exchange reaction can be selected if biomass optimization is the desired objective function. For modeling purposes, the species “biomass” is produced in the Yeast 5 GEM via a reaction which consumes 37 biomass precursor compounds to produce biomass, ADP, protons, and phosphate. The biomass precursors include water, polysaccharides, nucleotides, amino acids, riboflavin, sulfate, and the general species “lipid”. The “lipid” species in the Yeast 5 GEM serves a function similar to Zanghellini et al.’s “virtual membrane particle” [44] or Nookaew et al.’s lipid species [11]. It is a lumped species which incorporates many different specific lipid compounds. We have used two different definitions of “lipid” to enable simulation of aerobic and anaerobic biomass production (see “Simulating yeast growth”). The aerobic lipid pseudoreaction consumes 15 lipids and sterols, to produce the generic “lipid” species, while the anaerobic lipid pseudoreaction omits the sterols 14-demethyllanosterol and ergosta-5,7,22,24(28)-tetraen-3beta-ol. Thus, there are two Yeast 5 GEM biomass definitions: an aerobic biomass consisting of 52 compounds, and an anaerobic biomass definition consisting of 50 compounds. The growth, biomass pseudoreaction, and lipid pseudoreactions are detailed in Additional file 11: Table S6.

### Constraining reactions in the yeast 5 GEM

The Yeast 5 GEM has over 300 more directionally constrained reactions than Yeast 4. Such constraints incorporate thermodynamic information into the GEM, and often serve in part to limit Type III cycling, which arises from the linear programing approach, but are thermodynamically infeasible [45]. It is noted that such cycling can also be eliminated by minimizing the total flux, or by applying geometric FBA [46]. New constraints were added by re-evaluating “isa” reactions and applying a heuristic approach to reactions involving energy-carrying cofactors (ATP and NAD(P)), supported by evidence from other models, literature, and pathway databases. As discussed in “Generic demand reactions in Yeast 5 - towards a functional biomass definition”, directional constraints were added to “isa” reactions in the Yeast 5 GEM to prevent unrealistic interconversion of chemically distinct metabolites via fluxes through general species. Additional constraints were added with a heuristic approach similar to [47] which focuses on reactions which may produce ATP and those that use NAD or NADP as cofactors. We directionally constrained such reactions only when such constraints were supported by constraints in the iND750 and iMM904 reconstructions and by the directionality specified in the BioCyc database [5].

### Simulating yeast growth

The Yeast 5 GEM includes 170 exchange reactions, each of which defines a compound that can be included as a medium component for simulation purposes. As distributed, the simulated media is a glucose-limited minimal aerobic medium with constrained limited uptake of glucose, and unconstrained exchange of oxygen, ammonium, protons, iron(2+), phosphate, potassium, sodium, sulfate and water. To simulate anaerobic growth, the oxygen exchange reaction may be constrained to disallow oxygen uptake. Simulating anaerobic growth also requires that the simulated media be supplemented by allowing exchange of ergosterol, lanosterol, zymosterol and phosphatidate, and the biomass definition be changed by removing 14-demethyllanosterol and ergosta-5,7,22,24(28)-tetraen-3beta-ol from the “lipid” definition. These requirements reflect the observation that yeasts require sterols [48,49] and fatty acids [50] when cultured under rigidly anaerobic conditions. However, from a modeling perspective, these requirements arise from the biomass definition, for which the biochemistry is not firmly established, and from the reconstruction of sterol metabolism, which is incomplete in the Yeast 5 GEM.

Growth simulations were performed using the COBRA toolbox [26]. We have included MATLAB scripts which demonstrate simulation of aerobic and anaerobic growth, investigation of internal fluxes, and gene essentiality tests as supplemental material.

### Gene deletion simulation

Gene deletion simulations were performed using the COBRA toolbox [26]. To compare model gene essentiality predictions with observed phenotypes, gene lists were compiled for genes considered essential, and those which have been observed to cause auxotrophies when deleted. The essential gene list was compiled beginning with 1191 unique open reading frames reported to cause inviable mutants upon deletion in the Saccharomyces Genome Deletion Project [30] and the YKOv2 supplemental data set available from http://www-sequence.stanford.edu/group/yeast_deletion_project/data_sets.html. Since this data set screened in complex media (which is only incompletely accounted for with FBA simulation due to the limited number of exchange reactions), we refined the list of “essential” genes first by removing any ORFs which have not been reported as verified in the SGD database [28], and then by supplementing it with a list of gene mutations which have been reported to cause auxotroph phenotypes. The auxotroph-inducing gene list was generated by by searching the SGD database for “inviable” and “auxotrophy” phenotypes. Since the biochemistry of temperature signaling is beyond the scope of the Yeast 5 GEM, we removed temperature-dependent inositol auxotroph mutants [39] from this list. The lists of genes we used for evaluating model essentiality predictions, as well as a MATLAB script which can be used to evaluate other yeast GEMs essentiality predictions, are included as supplemental material.

### Simulation of genetic interactions

Genetic interactions were quantified with the nonscaled multiplicative definition of epistasis, ϵ = W_xy_-W_x_W_y_[35]_._ In this definition, W_x_ and W_y_ are fitness scores for organisms with a mutation in genes x and y, respectively, and W_xy_ is the fitness of the organism with both mutations present. Any ϵ ≠ 0 indicates a genetic interaction under the assumption that both of the genes independently and multiplicatively contribute to fitness. Following the example of [32], we quantified fitness as maximum biomass production rate (as determined by FBA) relative to the rate of biomass production in simulations conducted with the wild-type model. As suggested by [35], we simulated genetic perturbation by limiting flux through all reactions associated to a specific enzyme by a fixed amount of the wild-type geometric FBA flux (0%, 10%, 90%). Constraining the flux to fractions of wild-type flux allows investigation of essential reactions, as well as inessential reactions. The number of epistatic interactions for each level of flux restriction is included in Additional file 10: Table S5.

### MATLAB Scripts

modelToReconstruction.m: A script which can be used to generate the Yeast GENRE from the Yeast GEM; testYeastModel.m.m.; A script which can be used to compare various yeast GEMs (tested with iND750, iMM904, Yeast 4, and Yeast 5); fluxDistribution.m: A script demonstrating aerobic and anaerobic growth simulations, and comparing the fluxes through glycolysis, the TCA cycle, and the pentose phosphate pathway.

### SBML network files

yeast_5.01_model.xml: The Yeast GEM; yeast_5.01_recon.xml: The Yeast GENRE.

## MATLAB Scripts

model To Reconstruction.m, A script which can be used to generate the Yeast GENRE from the Yeast GEM; test Yeast Model.m, A script which can be used to compare various yeast GEMs (tested with iND750, iMM904, Yeast 4, and Yeast 5); flux Distribution. m, A script demonstrating aerobic and anaerobic growth simulations, and comparing the fluxes through glycolysis, the TCA cycle, and the pentose phosphate pathway; yeast_5.01_model.xml, The Yeast GEM; yeast_5.01_recon.xml, The Yeast GENRE.

## SBML network files

model To Reconstruction.m, A script which can be used to generate the Yeast GENRE from the Yeast GEM; test Yeast Model.m, A script which can be used to compare various yeast GEMs (tested with iND750, iMM904, Yeast 4, and Yeast 5); flux Distribution. m, A script demonstrating aerobic and anaerobic growth simulations, and comparing the fluxes through glycolysis, the TCA cycle, and the pentose phosphate pathway; yeast_5.01_model.xml, The Yeast GEM; yeast_5.01_recon.xml, The Yeast GENRE.

## Competing interests

The authors declare that they have no competing interests.

## Authors’ contributions

BDH contributed sphingolipid updates to the GENRE, added constraints to improve GEM flux predictions, compiled the essential and auxotroph-inducing gene lists, and wrote the manuscript. KS conducted the FBA for model evaluation and comparison, wrote the Matlab scripts included as supplemental data, and maintains the Yeast SBML and database files. BB conducted the epistasis analysis. PM leads the Computational Systems Biology & Biochemical Networks Modeling Group at the University of Manchester. LPW leads the Biomass Conversion Group at Cornell University. All authors have contributed to writing and have approved the manuscript.

## Supplementary Material

Additional file 1 Function testYeastModel.m.m. Click here for file

Additional file 2 Function modelToReconstruction.m. Click here for file

Additional file 3 Function fluxDistribution.m. Click here for file

Additional file 4 Yeast metabolic network reconstruction. Click here for file

Additional file 5 Yeast metabolic network model. Click here for file

Additional file 6 **Table S1.** ORFs in Yeast 4 not in Yeast 5. Click here for file

Additional file 7 **Table S2.** ORFs in Yeast 5 not in Yeast 4. Click here for file

Additional file 8 **Table S3.**Genes considered essential. Click here for file

Additional file 11 **Table S6.** Biomass Definition. Click here for file

Additional file 9 **Table S4.** Auxotroph-inducing genes. Click here for file

Additional file 10 **Table S5.** Gene interaction analysis. Click here for file
